# Morphology of intervertebral disc ruptures evaluated by vacuum phenomenon using multi-detector computed tomography: association with lumbar disc degeneration and canal stenosis

**DOI:** 10.1186/s12891-018-2086-7

**Published:** 2018-05-24

**Authors:** Koichiro Murata, Koji Akeda, Norihiko Takegami, Kevin Cheng, Koichi Masuda, Akihiro Sudo

**Affiliations:** 1 Department of Orthopaedic Surgery, Mie University Graduate School of Medicine, 2-174 Edobashi, Tsu City, Mie 514-8507 Japan; 2 Department of Orthopaedic Surgery, University of California, San Diego, 9500 Gilman Dr, La Jolla, CA 92093-0863 USA

**Keywords:** Intervertebral disc, Intervertebral disc degeneration, Intervertebral disc vacuum phenomenon, Computed tomography, Lumbar spinal stenosis

## Abstract

**Background:**

The progression of intervertebral disc (IVD) degeneration leads to rupture within IVD tissues. The location and appearance of areas of gaseous radiolucency in the IVD, known as vacuum phenomena (VPs), are considered to indirectly indicate the position and extent of IVD rupture. The clinical significance of VPs in degenerated IVDs is not fully understood. The purpose of this study is to assess and classify the morphology of IVD ruptures by the presence of intradiscal VPs, and to examine the association between morphological VP-positive IVD ruptures and degenerative lumbar diseases.

**Methods:**

IVD rupture was evaluated by the presence of VPs using computed tomography (CT) imaging. VP shape (spot, linear, island) was classified using sagittal imaging, and VP distribution (A-N: anterior AF-NP; N: NP only; N-P: NP-posterior AF; A-N-P: anterior and posterior AF-NP) was classified using axial imaging. The disc height index (DHI) was calculated from lateral radiographs. Disc degeneration and lumbar spinal stenosis were evaluated by MRI grade.

**Results:**

In the VP shape analysis, the island type was the most common, followed by linear and spot types. In the VP distribution analysis, A-N was the most common group, followed by N, N-P and A-N-P. Intra- and inter-observer reliabilities were statistically sufficient to classify different rupture shapes and distributions. The DHI tended to be lower in discs that contained VPs, especially in the anterior AF area. The shape and distribution of intradiscal VPs were significantly associated with the degree of disc degeneration and lumbar spinal stenosis graded by MRI. Discs with VPs extending from the NP into the anterior and/or posterior AF had a significantly higher proportion of advanced disc degeneration (Pfirrmann’s classification: grades IV and V).

**Conclusions:**

This is the first study to analyze the morphology of IVD rupture evaluated by the presence of intradiscal VPs using CT imaging. This classification can comprehensively present the shape and axial distribution of VPs within IVDs. Intradiscal VPs are associated with the progression of disc degeneration and lumbar spinal stenosis.

## Background

The intervertebral disc (IVD) consists of an outer annulus fibrosus (AF), which is rich in collagens accounting for its tensile strength, and an inner nucleus pulposus (NP), which contains large proteoglycans that retain water to maintain the osmotic pressure required for resistance against loading by compression. IVD degeneration is biochemically characterized by decreases in proteoglycan and water content in the NP, as well as collagen degeneration in the AF. The progression of IVD degeneration is known to lead to ruptures (including tears and/or cleft formation) within IVD tissues [1, 2].

Several authors have reported the morphological features of IVD ruptures [1–3], and have suggested the possibility that disc ruptures may be related to low back pain and/or the pathogenesis of degenerative lumbar diseases. IVD vacuum phenomena (VPs) refer to the radiographic appearance of a lucency caused by the presence of gas, usually in the lumbar region; this is one of the characteristics of IVD degeneration [4]. The location and appearance of gas is considered to indirectly indicate the position and extent of rupture within the IVD. However, only a few studies have reported the clinical relevance of intradiscal VPs [5, 6]. There have been no reports evaluating the types and distribution of intradiscal VPs or the association of intradiscal VPs and disc degeneration and/or degenerative disc diseases. Although magnetic resonance imaging (MRI) plays an important role in evaluating IVD pathologies [7], including tissue degeneration, it is less accurate than computed tomography (CT) for detecting VPs [8].

The purposes of this study were (1) to evaluate and classify the morphology of IVD rupture by the presence of VPs using multi-detector computed tomography (MDCT) imaging, and (2) to examine the association between morphological IVD rupture and radiological lumbar disc degeneration and canal stenosis.

## Methods

### Subjects

This IRB-approved retrospective study was conducted on spinal computed tomography (CT) images of 99 consecutive patients (48 men and 51 women) who underwent spinal surgery. The overall average age of the patients was 67.5 years-old (range, 23–90). A total of 590 discs from T12/L1 to L5/S1 were analyzed. The clinical diagnoses of the patients were as follows: 74 lumbar spinal stenosis (LSS), 15 lumbar disc herniation (LDH), 7 cervical spinal diseases, and 3 others (Table 1). Multi-detector CT (MDCT) (slice increment: 1.0 mm, slice thickness: 1.0 mm; Asteion TSX-021B, Toshiba Medical Systems Co., Otawara, Tochigi, Japan) was performed for all patients. Lateral lumbar spine radiography was also performed in 75 patients and magnetic resonance imaging (MRI) in 74 patients. All radiological images were taken within three-months before surgery.

**Table 1:** Patient demography

Diagnosis	Subjects (male/female)	Average age
LSS	74 (34/40)	71.5 (23–90)
LDH	15 (9/6)	53.6 (36–77)
Cervical diseases	7 (6/1)	62 (44–88)
Others	3 (2/1)	61.3 (50–67)
Total	99 (51/48)	67.5 (23–90)

Computed tomography (CT) images of 99 patients (51 men and 48 women) with spinal diseases were reviewed. The overall average age was 67.5 years-old (range: 23–90). The clinical diagnoses of the patients were 74 lumbar spinal stenosis (LSS), 15 lumbar disc herniation (LDH), 7 cervical spinal diseases, and 3 others

### Morphological classification of VPs

Intradiscal VPs were evaluated by the presence of areas of gaseous radiolucency using MDCT imaging. The morphological classification of intradiscal VPs included two separate categories, VP shape and rupture distribution.

### VP shape

**Fig. 1:**
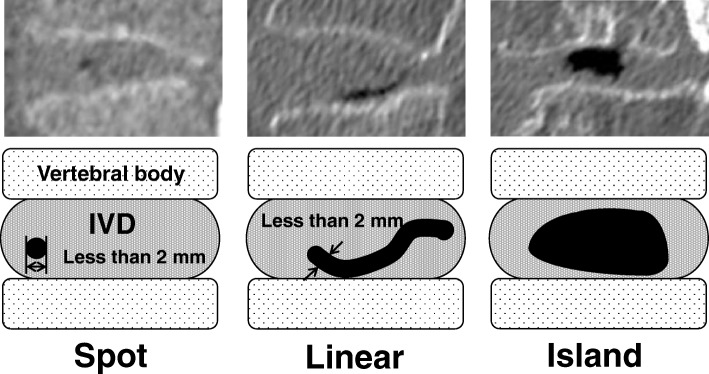
Shape type of vacuum phenomena. The presence of vacuum phenomena (VPs) was classified into three types (spot, linear, and island) using sagittal imaging by multi-detector computed tomography (MDCT)

VP shape was evaluated using mid-sagittal imaging (Fig. 1). VP shapes were categorized according to three classifications: spot, linear, and island. A spot-type VP was defined as a point-like VP less than 2 mm in diameter. A linear-type rupture was defined as a radiating VP whose width was less than 2 mm. An island-type rupture was defined as a VP forming a wide cleft (> 2 mm).

### VP distribution

**Fig. 2:**
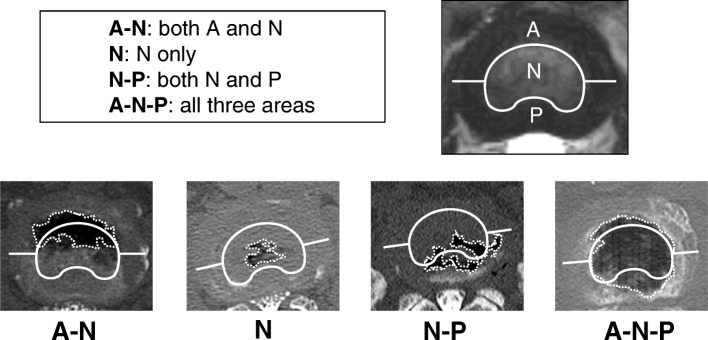
Distribution of vacuum phenomena. The distribution of vacuum phenomena (VPs) was classified using axial imaging by multi-detector computed tomography (MDCT). The intervertebral disc (IVD) was divided into three areas (nucleus pulposus: N; anterior annulus fibrosus: A; and posterior annulus fibrosus: P). Then, the distribution of intradiscal VPs was classified into four groups using combinations of these areas

VP distribution was classified using mid-axial imaging of intervertebral space (Fig. 2). The IVD was divided into three areas: nucleus pulposus (N), anterior annulus fibrosus (A), and posterior annulus fibrosus (P) on axial CT images of the disc. Using these divisions, the distribution of the intradiscal VPs was classified into four categories created by combinations of the three areas: 1) **A-N** indicates a VP in both the anterior annulus fibrosis and nucleus pulposus, 2) **N** indicates a VP in only the nucleus pulposus, 3) **N-P** indicates a VP in both the nucleus pulposus and the posterior annulus fibrosis, and 4) **A-N-P** indicates a VP in the anterior and posterior annulus fibrosus as well as the nucleus pulposus.

### Disc height index (DHI)

Using lateral lumbar spine radiographs of each subject, anterior disc height (*Ha*), posterior disc height (*Hp*), superior disc depth (*Ds*) and inferior disc depth (*Di*) were measured. Disc height was expressed as the DHI, which was calculated as: [(*Ha* + *Hp*) / (*Ds* + *Di*)] × 100 [9].

### Classification of disc degeneration

The degree of disc degeneration was evaluated by sagittal T2-weighted lumbar MRI, and was graded according to Pfirrmann’s classification from Grades I to V [10]. Grades IV and V were defined as advanced stages of IVD degeneration.

### Classification of lumbar spinal stenosis (LSS)

The extent of lumbar spinal stenosis was graded from A to D using the method based on the morphologic appearance of the dural sac (cerebrospinal fluid / rootlet ratio) on T2-weighted axial imaging [11]. Grades C and D were defined as severe stenosis.

### Data analysis

Two observers (orthopaedic surgeons) assessed the CT classifications of VP shape and distribution. The reliability of the CT classifications using CT images from 55 randomly selected VP-positive discs (52 patients) was evaluated using agreement percentage and kappa statistics for both intra- and inter-observers [12]. The agreement was regarded as “substantial” when the kappa values were more than 0.61 [13].

### Statistical analysis

Differences in the proportion of CT-classifications between males and females, and among age groups were statistically analyzed using the chi-square test or Fisher’s exact test. Statistically significant differences between the presence of VPs and DHI were assessed using one-way ANOVA followed by post hoc multiple comparisons using the Bonferroni method. The association between the morphology of ruptures (shape and distribution) and the MRI grading of disc degeneration or lumbar spinal stenosis was statistically assessed by the Fisher’s exact test followed by a post hoc test. The post hoc test was performed to assess the probability values for each combination of independent category levels by using a Bonferroni correction to control for type I error inflation [14, 15]. All the statistical analyses were performed using IBM SPSS Statistics (IBM Japan, Tokyo or IBM Corp., Armonk, NY, USA).

## Results

**Fig. 3:**
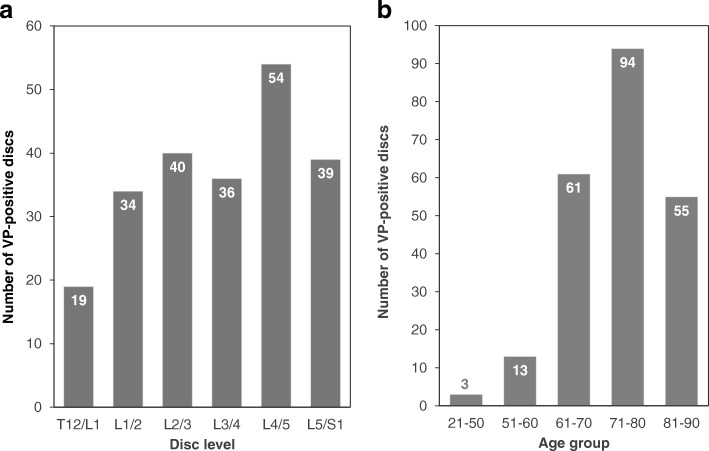
Number of vacuum phenomena (VP)-positive discs at different intervertebral disc levels (**a**) and by different age groups (**b**)

A VP was found in 226 discs (38.3%) of the 590 discs analyzed. The number of VP-positive discs was highest in the L4/5 level and lowest in the T12/L1 level (n [% of total VP-positive discs]: T12/L1: 19 [8.4%]; L1/2: 34 [15.0%]; L2/3: 40 [17.7%]; L3/4: 36 [16.0%]; L4/5: 54 [23.9%]; L5/S1: 43 [19.0%]) (Fig. 3a). The number of VPs was highest in the seventy (71–80 years-old) age group, followed by eighty, sixty, fifty and under fifty age groups (% of total VP-positive discs: 21–50 years-old: 3 [1.3%], 51–60: 13 [5.8%]; 61–70: 61 [27.0%]; 71–80: 93 [41.6%]; 81–90: 55 [24.3%]) (Fig. 3b). The percentage of VP-positive discs by age group was highest in the eighty age group, followed by seventy, sixty, fifty and under fifty age groups (% of VP-positive discs by age group: 21–50 years-old: 4.6%, 51–60: 21.7%; 61–70: 35.7%; 71–80: 46.1%; 81–90: 61.1%).

### Intra- and inter-observer agreement of VP shape type

Intra- and inter-observer agreement of the VP shape type was “substantial” with kappa values 0.75–0.76 and 0.67, and with % agreements 87.3–89.1% and 83.6, respectively. In the VP distribution analysis, intra- and inter-observer agreement was also “substantial” with kappa values 0.64–0.65 and 0.70, and with % agreements 74.5–76.4 and 80.0%, respectively.

### Prevalence of various VP types and distribution

**Fig. 4:**
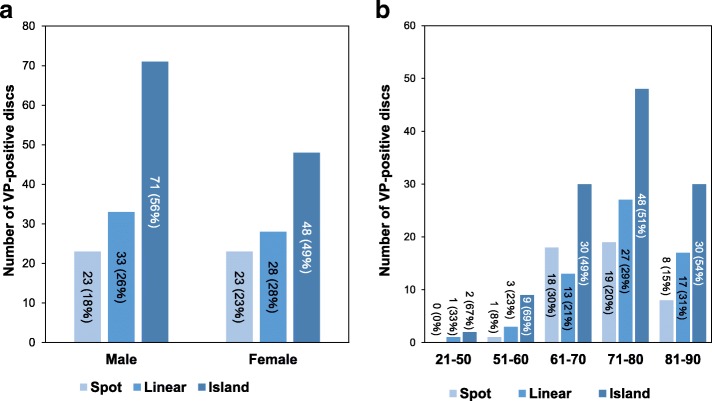
Characteristics of intradiscal vacuum phenomena (VPs) in shape classification. **a** Number of vacuum phenomena (VPs) positive-discs by shape classification in males and females. **b** Number of VP-positive discs by age group by VP shape classification. The counts of VP-positive discs are written in the graph. Percentages in parentheses indicate the proportion of VP-positive discs of each rupture shape compared with the total number of VP-positive discs by gender (**a**) and by age group (**b**)

VP shape type analysis revealed that among the total number of VP-positive discs, the island type was the most common (119 discs [53%]), followed by linear (61 discs [27%]) and spot (46 discs [20%]). There was no significant difference in the proportion of each VP shape between males (Spot: 18%; Linear: 26%; Island 56%) and females (Spot: 23%; Linear: 28%; Island 49%) (*P* = 0.47) (Fig. 4a). No significant differences in these proportions were identified among all age groups (*P* = 0.5), and the island type had the highest proportion in all age groups more than 51 years-old (Fig. 4b).

**Fig. 5:**
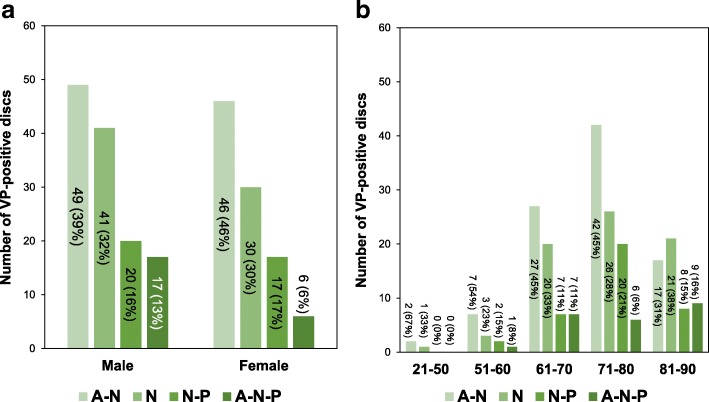
Characteristics of intradiscal vacuum phenomena by distribution. **a** Number of vacuum phenomenon (VP)-positive discs in males and females. **b** Number of vacuum phenomena (VPs) by age group in VPs distribution. The counts of VP-positive discs are written in the graph. Percentages in parentheses indicate the proportion of VP-positive discs of each rupture shape compared with the total number of VP-positive discs by gender (**a**) and by age group (**b**). VP: vacuum phenomenon; VP-: non-ruptured group; Nucleus pulposus: N; anterior annulus fibrosus: A; and posterior annulus fibrosus: P

The analysis of VP distribution revealed that A-N was the most common area group (95 discs [42%]), followed by N (71 discs [32%]), N-P (37 discs [17%]) and A-N-P (23 discs [9%]) area groups. There were no significant differences in the proportion of each VP distribution between males (A-N: 39%; N: 32%; N-P: 16%; A-N-P: 13%) and females (A-N: 46%; N: 30%; N-P: 17%; A-N-P: 6%) (*P* = 0.23) (Fig. 5a). No significant differences in these proportions were found in all age groups (*P* = 0.52) (Fig. 5b).

### Association between the shape and distribution of VPs

A Pearson’s chi-square test revealed a significant association between the shape and distribution of VPs (*P* < 0.0001).

**Fig. 6:**
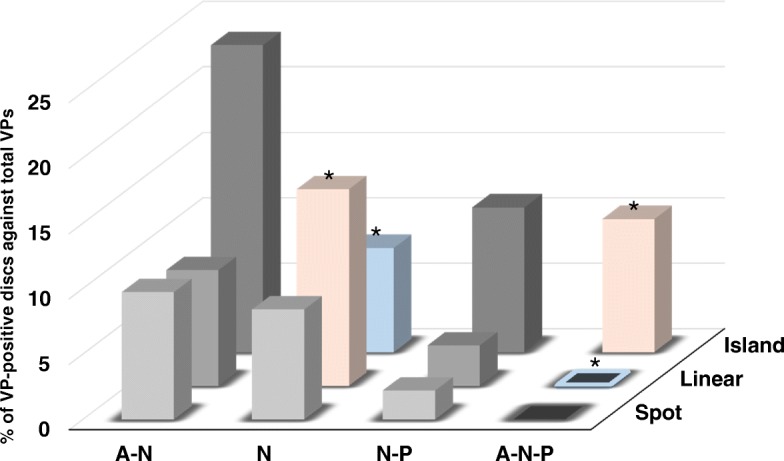
Association between vacuum phenomenon shape type and distribution. The percentage of vacuum phenomenon (VP)-positive discs of the total number of VP-positive discs in each combination of shape and distribution classification is presented. Nucleus pulposus: N; anterior annulus fibrosus: A; and posterior annulus fibrosus: P. *P < 0.004 (by Bonferroni correction)

Spot type VPs tended to be distributed in the A-N (% of total number of VP-positive discs: 9.7%) and N (8.4%) areas with high frequency; however no significant differences were identified in this distribution trend (Fig. 6). Linear type VPs were most highly distributed in the N (15.0%) area with statistical significance (*P* < 0.0001), followed by A-N (8.8%), then N-P (3.1%) areas (Fig. 6). No linear type VPs were found in the A-N-P area (*P* < 0.0001). Island type VPs were most highly distributed in the A-N (23.5%) area, however no statistical difference was identified (*P* = 0.8). There was a significantly lower frequency of the island type located in the N area (8.0%, P < 0.0001), and a significantly higher frequency in the A-N-P area (10.2%, *P* < 0.004).

### Association of VP type and distribution with disc height

**Fig. 7:**
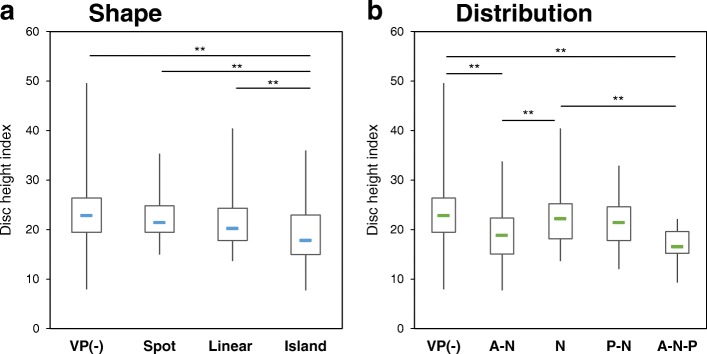
Association of disc height with shape type of vacuum phenomenon. **a** Shape type analysis. The disc height index (DHI) in the island type group was significantly lower than that of the other groups. **P < 0.01. **b** Distribution analysis. The DHI tended to be lower in the areas including the anterior AF. **P < 0.01. VP: vacuum phenomenon; VP-: non-ruptured group; Nucleus pulposus: N; anterior annulus fibrosus: A; and posterior annulus fibrosus: P

In the VP shape type analysis (Fig. 7a), the DHI of the VP-negative (−) group was significantly higher than that of the island type group (*P* < 0.01); however no significant differences were found with linear and spot types. Among the VP-positive (+) discs, the DHI tended to be highest in spot type VPs, lower in linear VPs, and lowest in island VPs. The DHI in the island type groups was significantly lower than that of the other groups (*P* < 0.01) (Fig. 7a).

In the VP distribution study (Fig. 7b), the DHI in A-N and A-N-P area groups was significantly lower than that of the VP (−) group (*P* < 0.01, respectively; Fig. 7b). In addition, the DHI in A-N and A-N-P area groups was significantly lower than that of the N distribution (*P* < 0.01).

### Association of VP shape type and distribution with disc degeneration

**Fig. 8:**
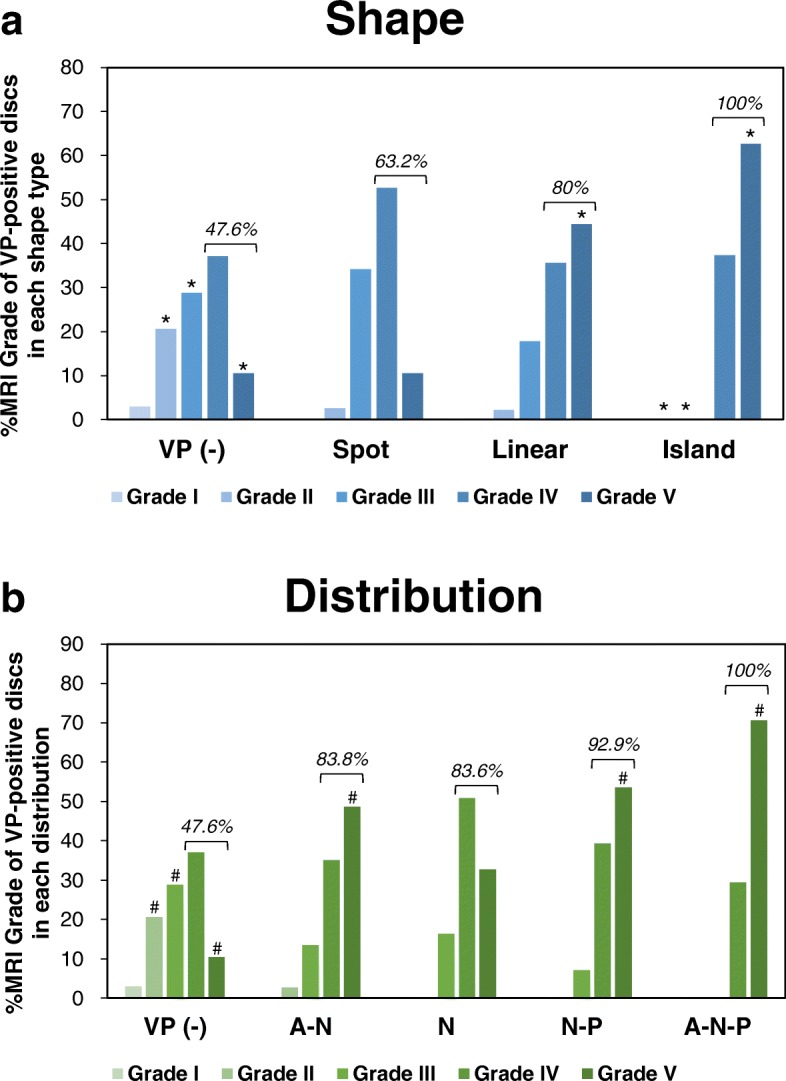
Percentage of MRI grade of disc degeneration in vacuum phenomenon shape type and distribution analyses. The percentage of MRI grade (Pfirrmann’s classification [10]) of disc degeneration in vacuum phenomenon (VP) shape type (**a**) and distribution (**b**) analyses. *P < 0.0025, ^#^*P* < 0.002 (by Bonferroni correction). The percentage of advanced degenerative stage discs (Pfirrmann grades IV and V) of each group is indicated by a bracket

In the VP shape type analysis (Table 2A), the Fisher’s exact test showed a significant association between VP shapes and MRI grades of disc degeneration (*P* < 0.0001). The results of the post-hoc test showed that VP (−) discs had a significantly higher proportion of discs with MRI grade 2 (percentages of the raw marginal total: 20.6%) and grade 3 (28.8%), but significantly lower with grade 5 (10.5%) (Table 2A, Fig. 8a). The proportion of spot type discs was highest in MRI grade 4 (53.6%); however those discs showed no significant association with MRI grading (Table 2A, Fig. 8a). In the linear type, the proportion of discs with MRI grade 5 (44.4%) was highest and significantly higher than other MRI grades (*P* < 0.0001). Island type discs had a significantly lower proportion of discs with MRI grade 2 (0%) and grade 3 (0%), but significantly higher with MRI grade 5 (62.6%) (*P* < 0.0001, respectively) (Table 2A, Fig. 8a).

**Table 2:** Association between intradiscal vacuum phenomena (VPs) and disc degeneration

A: Shape		MRI Pfirrmann’s classification of disc degeneration					Total
		I	II	III	IV	V	
VP (−)	Count (% raw)	8 (3.0%)	55 * **(20.6%)**	77 * **(28.8%)**	99 (37.1%)	28 * **(10.5%)**	267
	Exp. Count	4.8	**34.5**	**59.3**	102.3	**66.0**	
	Corrected *P*-value	0.02	**< 0.0001**	**< 0.0001**	0.51	**< 0.0001**	
Spot	Count(% raw)	0 (0%)	1 (2.6%)	13 (34.2%)	20 (52.6%)	4 (10.5%)	38
	Exp. Count	0.7	4.9	8.4	14.6	9.4	
	Corrected *P*-value	0.38	0.05	0.06	0.06	0.03	
Linear	Count (% raw)	0 (0%)	1 (2.2%)	8 (17.8%)	16 (35.6%)	20 * **(44.4%)**	45
	Exp. Count	0.8	5.8	10.0	17.2	**11.1**	
	Count (% raw)	0.03	0.02	0.45	0.69	**0.0012**	
Island	Count (% raw)	0 (0%)	0 * **(0%)**	0 * **(0%)**	34 (37.4%)	57 * **(62.6%)**	91
	Exp. Count	1.7	**11.8**	**20.2**	34.9	**22.5**	
	Corrected *P*-value	0.15	**< 0.0001**	**< 0.0001**	0.83	**< 0.0001**	
B: Distribution		MRI Pfirrmann’s classification of disc degeneration					Total
		I	II	III	IV	V	
VP (−)	Count (% raw)	8 (3.0%)	**55** ^**#**^ **(20.6%)**	**77** ^**#**^ **(28.8%)**	99 (37.1%)	**28** ^**#**^ **(10.5%)**	267
	Exp. Count	4.8	**34.5**	**59.3**	102.3	**66.0**	
	Corrected *P*-value	0.02	**< 0.0001**	**< 0.0001**	0.51	**< 0.0001**	
A-N	Count (% raw)	0 (0%)	2 (2.7%)	10 (13.5%)	26 (35.1%)	**36** ^**#**^ **(48.6%)**	74
	Exp. Count	1.3	9.6	16.4	28.4	**18.3**	
	Corrected *P*-value	0.20	0.004	0.45	0.53	**< 0.0001**	
N	Count (% raw)	0 (0%)	0 (0%)	9 (16.4%)	28 (50.9%)	18 (32.7%)	55
	Exp. Count	1.0	7.1	12.2	21.1	13.6	
	Corrected *P*-value	0.28	0.0023	0.26	0.04	0.14	
N-P	Count (% raw)	0 (0%)	0 (0%)	2 (7.1%)	11 (39.3%)	**15** ^**#**^ **(53.6%)**	28
	Exp. Count	0.5	3.6	6.2	10.7	**6.9**	
	Corrected *P*-value	0.46	0.004	0.05	0.91	**0.00025**	
A-N-P	Count (% raw)	0 (0%)	0 (0%)	0 (0%)	5 (29.4%)	**12** ^**#**^ **(70.6%)**	17
	Exp. Count	0.3	2.2	3.8	6.5	**4.2**	
	Corrected *P*-value	0.2	0.004	0.05	0.53	**< 0.0001**	

The results of a contingency-table test are presented. **A:** VP shape analysis. **B:** VP distribution analysis. Percentages of the raw marginal total (% raw) are in parentheses. Exp. Count: expected count; VP (−): non-ruptured group; Nucleus pulposus: N; anterior annulus fibrosus: A; and posterior annulus fibrosus: P. *P < 0.0025, ^#^*P* < 0.002 (by Bonferroni correction). The cells with counts above or below the expected count with statistical significance are shown in bold

The distribution of intradiscal VPs was also significantly associated with MRI grade (Fisher’s exact test, *P* < 0.0001) (Table 2B). The post-hoc test revealed that the discs with A-N, N-P and A-N-P distribution had the highest and significantly higher proportion of discs with MRI grade 5 (A-N: 48.6%, *P* < 0.0001; N-P: 53.6%, *P* < 0.002; A-N-P: 70.6%, *P* < 0.0001) than those with other MRI grades, respectively (Table 2B, Fig. 8b). Discs with N distribution were most frequently classified as MRI grade 4 (50.9%); however those discs showed no significant effect on the proportion of discs with different MRI grades (Table 2B, Fig. 8b).

Of all the discs analyzed by MRI (441 discs), 63.0% (278 discs) were in the advanced degenerative stage (Pfirrmann grades IV and V). In the VP shape type analysis, the percentage of discs with advanced stage of degeneration was highest in the island type (100%) followed by linear (80%) and spot (63.2%) types (Fig. 8a). In the VP distribution analysis, the discs with advanced stage of degeneration were most frequently distributed in the A-N-P (100%) area, followed by N-P (92.9%), A-N (83.8%) and N (83.6%) areas (Fig. 8b).

### Association of VP type and distribution with lumbar spinal stenosis

**Fig. 9:**
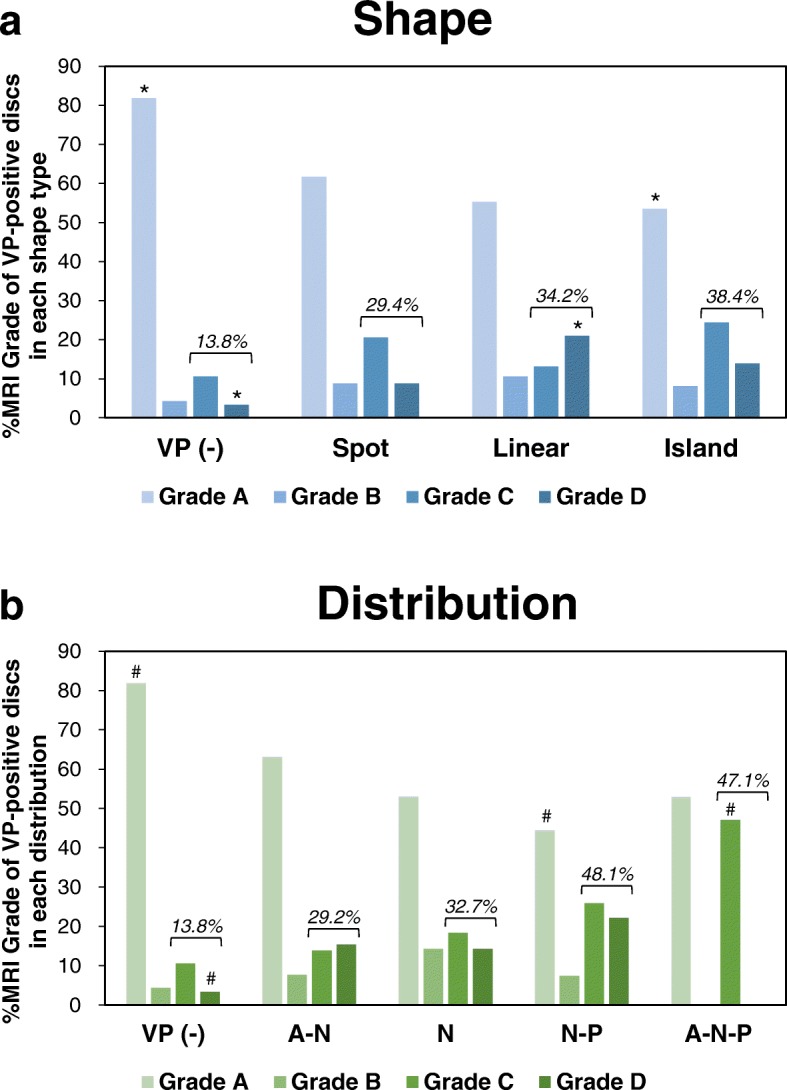
Percentage of MRI grade of lumbar spinal stenosis in vacuum phenomenon shape type and distribution analyses. The percentage of MRI grade (Schizas’s classification [11]) of lumbar spinal stenosis in vacuum phenomenon (VP) shape type (**a**) and distribution (**b**) analyses. *P < 0.0025, ^#^*P* < 0.002 (by Bonferroni correction). The percentage of VP-positive discs with severe stenosis (grades C and D) of each group is indicated by a bracket

The Fisher’s exact test showed a significant association between VP shape type and MRI grade of canal stenosis (*P* < 0.0001) (Table 3A). VP (−) discs had a significantly higher proportion of grade A stenosis (percentages of the raw marginal total: 81.8%), and significantly lower proportion of grade D stenosis (3.3%) at corresponding disc levels (Table 3A, Fig. 9a). Discs with a linear type VP had a significantly higher proportion of grade D stenosis (21.1%), and discs with an island type VP had a significantly lower proportion of grade A stenosis (53.5%) at corresponding disc levels (Table 3A, Fig. 9a).

**Table 3:** Association between intradiscal vacuum phenomena (VPs) and lumbar spinal stenosis

A: Shape		MRI classification of lumbar spinal stenosis				Total
		A	B	C	D	
VP (−)	Count (% raw)	171 * **(81.8%)**	9 (4.3%)	22 (10.5%)	7 * **(3.3%)**	209
	Exp. Count	**147.5**	13.1	31.3	**17.1**	
	Corrected *P*-value	**< 0.0001**	0.07	0.006	**< 0.0001**	
Spot	Count (% raw)	21 (61.8%)	3 (8.8%)	7 (20.6%)	3 (8.8%)	34
	Exp. Count	24.0	2.1	5.1	2.8	
	Corrected *P*-value	0.23	0.51	0.34	0.89	
Linear	Count (% raw)	21 (55.3%)	4 (10.5%)	5 (13.2%)	8 * **(21.1%)**	38
	Exp. Count	26.8	2.4	5.7	**3.1**	
	Corrected *P*-value	0.03	0.35	0.74	**0.0022**	
Island	Count (% raw)	46 * **(53.5%)**	7 (8.1%)	21 (24.4%)	12 (14.0%)	86
	Exp. Count	**60.7**	5.4	12.9	7.0	
	Corrected *P*-value	**0.0001**	0.41	0.005	0.025	
B: Distribution		MRI classification of lumbar spinal stenosis				Total
		A	B	C	D	
VP (−)	Count (% raw)	**171** ^**#**^ **(81.8%)**	9 (4.3%)	22 (10.5%)	**7** ^**#**^ **(3.3%)**	209
	Exp. Count	**147.5**	13.1	31.3	**17.1**	
	Corrected *P*-value	**< 0.0001**	0.07	0.006	**< 0.0001**	
A-N	Count (% raw)	41 (63.1%)	5 (7.7%)	9 (13.8%)	10 (15.4%)	65
	Exp. Count	45.9	4.1	9.7	5.3	
	Corrected *P*-value	0.14	0.60	0.77	0.02	
N	Count (% raw)	26 (53.1%)	7 (14.3%)	9 (18.4%)	7 (14.3%)	49
	Exp. Count	34.6	3.1	7.3	4.0	
	Corrected *P*-value	0.0039	0.013	0.48	0.09	
N-P	Count (% raw)	**12** ^**#**^ **(44.4%)**	2 (7.4%)	7 (25.9%)	6 (22.2%)	27
	Exp. Count	**19.1**	1.7	4.0	2.2	
	Corrected *P*-value	**0.0019**	0.07	0.005	0.0001	
A-N-P	Count (% raw)	9 (52.9%)	0 (0%)	**8** ^**#**^ **(47.1%)**	0 (0%)	17
	Exp. Count	12.0	1.1	**2.5**	1.4	
	Corrected *P*-value	0.1	0.27	**0.00015**	0.21	

The results of a contingency-table test are presented. **A:** VP shape analysis. **B:** VP distribution analysis. Percentages of the raw marginal total (% raw) are in parentheses. Exp. Count: expected count; VP (−): non-ruptured group; Nucleus pulposus: N; anterior annulus fibrosus: A; and posterior annulus fibrosus: P. *P < 0.0025, ^#^*P* < 0.002 (by Bonferroni correction). The cells with counts above or below the expected count with statistical significance are shown in bold

The distribution of VPs also had a significant association with the MRI grade of canal stenosis (Fisher’s exact test, *P* < 0.0001) (Table 3B). The discs with N-P distributions had a significantly lower proportion of MRI grade A canal stenosis (44.4%), and the discs with A-N-P distributions had a significantly higher proportion with MRI grade C canal stenosis (47.1%) at the corresponding disc levels (Table 3B, Fig. 9b). On the other hand, the discs with A-N and N distributions had no significant association with MRI grade of canal stenosis at corresponding disc levels (Table 3B, Fig. 9b).

The percentage of VP-positive discs with severe stenosis (grades C and D) at the corresponding disc level in all analyzed discs was 23.1%. The percentage of discs with severe stenosis in each shape type was highest in the island type (38.4%) followed by linear (34.2%) and spot (29.4%) types (Fig. 9a). The VP in discs with severe stenosis at the corresponding disc level was most frequently distributed to the N-P (48.1%) area, followed by A-N-P (47.1%), N (32.7%) and A-N (29.2%) areas (Fig. 9b).

## Discussion

To evaluate the morphology of IVD ruptures in the lumbar spine, we performed a cross-sectional retrospective CT study to assess intradiscal VPs of consecutive patients who underwent spinal surgery. The results showed that VPs were found in 38.3% of the 587 discs analyzed, most prominently at the L4/5 level and highest in the seventy years-old population. The type and distribution of intradiscal VPs were significantly associated with severity of MRI grade of disc degeneration and disc height narrowing. The presence of VPs within the disc also had a significant effect on the lumbar spinal stenosis of corresponding IVDs.

Intradiscal VPs are most commonly seen in patients with disc degeneration, especially in the NP, where dehydration of the disc and enlarging clefts lead to the accumulation of gas [4, 16, 17]. VPs in IVDs were first described by Magnusson in 1937 [18] and are a common finding that can be observed in 1–3% of healthy adults on spinal radiographs [19–21], but can reach a prevalence of 20% in elderly patients [4, 22, 23]. Ford and colleagues reported that intradiscal VPs contain 90–92% nitrogen combined with oxygen, carbon dioxide and traces of other gases [24]. Because nitrogen gas is hard to metabolize within tissues in vivo, it can accumulate within the tissue organization.

With the progression of disc degeneration, the crevices extend peripherally, affecting first the inner fibers and later the outer fibers of the AF. At the same time, gas collections may become more prominent, extending from the interior to the exterior of the affected disc [4]. Therefore, the position and form of intradiscal VPs are considered to indicate the distribution and extent of ruptures within IVD tissues. Gas accumulation in the spinal canal or in disc herniation is reported to be associated with neurologic symptoms, although the clinical significance of intradiscal VPs has been controversial.

In this study, we first classified the morphology of intradiscal VPs, using CT images to evaluate the degree of disc rupture by shape (sagittal-plane) and distribution (axial–plane). Both intra- and inter-observer agreements of this two-part classification system are “substantial” by kappa statistics, and showed higher % agreements, suggesting that this classification system would be sufficient to distinguish between the different VP shapes and distributions.

The results of our study showed evidence that the presence of intradiscal VPs has a significant association with the degree of disc degeneration graded by MRI. In addition, the extent of disc ruptures evaluated by the types and distribution of intradiscal VPs also significantly affects the MRI grading of disc degeneration. Discs with linear and island shape VPs had a significantly higher proportion of MRI grade V discs, which indicated the collapse of discs with significant structural changes [10]. In the distribution analysis, except for the N distribution, all the discs with the VPs that extend from NP into anterior and/or posterior AF (A-N, N-P, A-N-P) had a significantly higher proportion of MRI grade V discs. These results suggested that intradiscal VPs extending from the N into AF area are associated with significant structural changes of IVDs.

The results of lumbar radiography also showed that there is a significant trend for disc height to decrease in accordance with the extent of disc rupture evaluated by the type of intradiscal VPs. Taking the distribution of VPs into account, disc height was significantly lower in discs that contained VPs in the area of the anterior AF. In the clinical setting, disc height narrowing is the commonly used radiographic finding indicating disc degeneration; however, according to Pfirrmann’s classification [10], this change is recognized as a finding related to an advanced stage (more than grade IV) of disc degeneration with significant structural changes [9].

We also evaluated the association between intradiscal VPs and lumbar spinal stenosis. In this study, we also showed evidence that lumbar spinal stenosis was significantly associated with the presence of VPs at the corresponding IVD. Severe stenosis (grades C and D by MRI classification [11]) was most frequently identified in IVDs containing the island type VP, especially those including the posterior AF. Ligamentum flavum thickness, disc protrusion, and spondylolisthesis are major pathogeneses of lumbar spinal stenosis. Intradiscal VPs have been recognized as one of the signs of segmental instability [5, 25–27]. Therefore, the results of our study further support the possibility that the presence of intradiscal VPs would lead to segmental instability, which is responsible for the pathogenesis of lumbar spinal stenosis.

Clinical relevance: Recently, the effects of intradiscal VPs on the clinical (surgical) outcome of lumbar fusion surgery have been reported [5, 27, 28]. Liao et al. [5] reported that intradiscal VPs should be regarded as a sign of intervertebral instability. Hence, lumbar interbody fusion (LIF) surgery with intervertebral cage, but not posterolateral fusion only, should be performed for patients with degenerative lumbar spondylolisthesis containing intradiscal VPs. LIF surgery including lateral and posterior approaches with cages has been reported to be effective for restoration of disc height, lumbar lordosis and also for improving the bone fusion rate in lumbar spondylolisthesis patients with intradiscal VPs [5, 27, 28]. The results of this study also revealed that intradiscal VPs, especially those extending from the N into the AF region, were associated with structural destruction of IVD tissues, including disc height narrowing. Therefore, our morphological classification of VPs might be advantageous in determining the surgical procedure used for treating degenerative lumbar diseases.

Limitations of this study were that most of the subjects were patients who had taken pre-operative CT images for elective spinal surgeries, and that an age-matched general population was not used as a control in this study. Therefore, the percentage of intradiscal VPs in our subjects would be much higher than that within an age-matched general population. This is the first study analyzing the shape type and distribution of intradiscal VPs using CT images to indicate that VPs were frequently distributed in the NP and anterior AF. However, it should be kept in mind that the anterior distribution of VPs would be more enhanced in a CT study using a supine position compared with the exact distribution of disc ruptures evaluated in cadaveric studies [1, 2]. Another limitation was that the morphological classification of intradiscal VPs was performed using sagittal and axial CT images analyzed separately with different classifications. A three-dimensional analysis of VPs may be needed for more precise evaluations of area, distribution and volume of intradiscal VPs.

Importantly, when the intradiscal VPs were evaluated by MRI T2-wighted images, approximately 10% of VP-positive discs showed high signal intensity, demonstrating MR imaging findings similar to those of infectious spondylodiscitis [7, 29, 30]. The presence of intradiscal VPs evaluated by CT images would aid in the exclusion diagnosis of infectious spondylodiscitis [31].

## Conclusions

This study describes the assessment of IVD ruptures by the presence of intradiscal VPs using CT-imaging. This two-part classification can comprehensively present the shape and axial distribution of VPs within IVDs, with intra- and inter-observer reliability sufficient to classify different rupture shapes and distributions. This study also showed evidence that the presence of intradiscal VPs is associated with MRI-grading of disc degeneration and radiographic disc height narrowing. Therefore, the presence of intradiscal VPs represents one of the signs of disc ruptures with a significant structural change. This systematic analysis of intradiscal VPs also revealed that the shape and distribution of VPs were significantly associated with the MRI-grading of lumbar canal stenosis at the corresponding disc level. To further evaluate the clinical relevance of intradiscal VPs, it would be important in future studies to examine the association between segmental intervertebral instability and/or clinical symptoms using this CT-based analytical system.

## Abbreviations

AF: annulus fibrosus
CT: computed tomography
DHI: disc height index
IVD: intervertebral disc
LSS: lumbar spinal stenosis
MDCT: multi-detector computed tomography
MRI: magnetic resonance imaging
NP: nucleus pulposus
VP: vacuum phenomenon
